# Cross-cultural adaptation and psychometric properties of the Chinese version of the postpartum depression literacy scale

**DOI:** 10.3389/fpsyg.2022.966770

**Published:** 2022-08-09

**Authors:** Pingping Guo, Nianqi Cui, Minna Mao, Xuehui Zhang, Dandan Chen, Ping Xu, Xiaojuan Wang, Wei Zhang, Qiong Zheng, Le Zhang, Zhenzhen Xiang, Yin Jin, Suwen Feng

**Affiliations:** ^1^Women’s Hospital, Zhejiang University School of Medicine, Hangzhou, China; ^2^Faculty of Nursing, Zhejiang University School of Medicine, Hangzhou, China; ^3^School of Nursing, Jining Medical University, Jining, China

**Keywords:** postpartum depression literacy, perinatal women, cultural adaptation, psychometric properties, scale, Chinese

## Abstract

**Background and aim:**

The postpartum depression literacy (PoDLi) of perinatal women is closely related to the occurrence, recognition, and treatment of postpartum depression, therefore valid instruments for evaluating the level of PoDLi are of great significance for both research and clinical practice. This study aimed to cross-culturally adapt the postpartum depression literacy scale (PoDLiS) into Chinese and to test its psychometric properties among Chinese perinatal women.

**Materials and methods:**

A cross-sectional study was conducted from April to May 2022 in a tertiary hospital in Hangzhou, Zhejiang Province, China. 619 out of the 650 perinatal women that were approached *via* a convenience sampling method completed the Chinese version of the PoDLiS (C-PoDLiS). Content validity [the content validity index of items (I-CVI) and scale-level content validity index (S-CVI)] was evaluated by an expert panel. Psychometric properties, including item analysis, structure validity (exploratory factor analysis, confirmatory factor analysis), convergent and discriminant validity, reliability (internal consistency, test-retest reliability), criterion validity (concurrent validity, predictive validity), and floor/ceiling effect were examined.

**Results:**

The final version of C-PoDLiS is a six-factor structure consisting of 27 items, which explained 61.00% of the total variance. Adequate content validity (I-CVI = 0.833–1.00, S-CVI = 0.920) was ensured by the expert panel. The modified confirmatory factor analysis model revealed that the 6-factor model fitted the data well (χ^2^/*df* = 1.532, root mean square error of approximation = 0.042, goodness of fit = 0.900, incremental fit index = 0.949, comparative fit index = 0.948, Tucker–Lewis index = 0.940). The total Cronbach’s α was 0.862, the total McDonald’s ω was 0.869, and the test-retest reliability coefficient was 0.856. Results of convergent validity (average variance extracted = 0.486–0.722) and discriminant validity provided good or acceptable psychometric support. Significant correlations between scores of the C-PoDLiS and Mental health literacy scale (*r* = 0.155–0.581, *p* < 0.01) and Attitudes toward seeking professional psychological help short form scale (*r* = 0.361–0.432, *p* < 0.01) supported good concurrent and predictive validity, respectively. No floor/ceiling effect was found.

**Conclusion:**

The C-PoDLiS was demonstrated to be a sound instrument with good reliability and validity for evaluating Chinese perinatal women’s PoDLi levels. Its use in the future can facilitate data aggregation and outcome comparisons across different studies on this topic.

## Introduction

Depression has affected over 350 million people globally (World Health Organization [WHO], 2016). It is further predicted that by 2030, depression will be the leading cause of disease burden worldwide (World Health Organization [WHO], 2013; Bernstein et al., 2021). Women are nearly twice more likely to undergo depression across their lifespan than men (Albert, 2015), whereas the perinatal period appears to be a stage of higher risk for developing this disease due to biological changes as well as role transitions in family and society (Staneva et al., 2015; Bjelica et al., 2018). In particular, postpartum depression (PPD) is the most common psychiatric condition during the perinatal period (Evagorou et al., 2016), which is defined as a depressive episode with moderate to severe severity that occurs within the first 12 months after childbirth (O’Hara and McCabe, 2013) and is characterized by an inability to experience pleasure, spontaneous crying, appetite and sleep disturbance, fatigue, attention and concentration impairment, feelings of guilt and despair, and even thoughts of suicide (American Psychiatric Association, 2013). The reported worldwide prevalence statistics of PPD varied widely [ranging from 3.2 to 63.3% (Evagorou et al., 2016)] as a consequence of differences in sample population, instruments, diagnostic criteria, and the time point of screening, with an overall prevalence of 17.7% (Hahn-Holbrook and Cornwell-Hinrichs, 2018) and a major PPD prevalence of 6% (Ashley et al., 2016). Furthermore, research showed that these rates may be even higher in vulnerable groups of women (e.g., racial and ethnic minorities, adolescents, and low socioeconomic status) (Pooler et al., 2013) and in developing regions (Husain et al., 2006; Özcan et al., 2017). Actually, PPD has become one of the greatest unaddressed global challenges of the 21st century (Rahman et al., 2013). PPD can lead to a great number of short-term and long-term negative consequences for the mother, baby, and family without timely and effective management (O’Hara and McCabe, 2013; Field, 2018), which include but are not limited to increased risks of suicide and future episodes of depression in the mother, earlier discontinuation of breastfeeding, impaired mother-child bonding, increased economic costs, and tensions and broken relationships between family members. Noteworthily, these adverse effects can be minimized with effective treatments (Williams et al., 2016). Hence, early detection and efficient treatment of PPD are quite essential.

Unfortunately, the existing evidence revealed an unsatisfactory situation in the recognition and treatment of PPD (McCarthy and McMahon, 2008; Bilszta et al., 2010; Ko et al., 2012). On the one hand, since no procedure for standardizing PPD screening has been established in the majority of countries around the world, the identification of PPD is largely dependent on women×s knowledge of the associated symptomatology (Fonseca et al., 2015). However, the phenomenon is often that women report having limited knowledge about PPD. As such, they have difficulties distinguishing between the normative distress associated with the transition to parenthood and the PPD symptoms (Bilszta et al., 2010), as well as assessing the severity of the PPD symptoms (McCarthy and McMahon, 2008). Research showed that only about 40% of PPD cases are diagnosed (Ko et al., 2012). On the other hand, in spite of the availability of effective psychological and pharmacological treatment strategies, the vast majority of women are less likely to engage in professional help-seeking for PPD and proactively disclose their symptoms (Fonseca et al., 2015). According to studies, less than half of women with PPD sought professional assistance or engaged in standard treatments (Dennis and Chung-Lee, 2006; Sharma and Sharma, 2012; Geier et al., 2015). To date, two categories of barriers that may prevent women from seeking professional help for PPD have been identified (Dennis and Chung-Lee, 2006), including system-related barriers that are relatively difficult to change and pertain to circumstances beyond the individual’s control (e.g., childcare responsibilities, work constraints, inconvenient traffic, financial difficulties, inequal health resource distribution) and individual-level barriers (e.g., poor awareness concerning PPD, confusion related to the symptoms and treatments of PPD, feelings of guilt, shame, and stigma about PPD). The individual-level barriers, more explicitly, are the shortage of knowledge and the inaccurate views and perceptions regarding PPD (Dennis and Chung-Lee, 2006; Fonseca et al., 2015), which is called “low postpartum depression literacy (PoDLi)” (Fonseca et al., 2017) and has attracted much attention in recent years due to its modifiability and improveability.

Postpartum depression literacy is essentially a subset of mental health literacy (MHL), referring to knowledge, attitude, and beliefs about PPD as well as the ability to make informed treatment decisions (Mirsalimi et al., 2020). The most classic and comprehensive conceptual framework of MHL was introduced by Jorm (2000), which encompasses the following six components: (i) the ability to recognize different types of mental health disorders; (ii) knowledge and beliefs regarding the causes and risk factors; (iii) knowledge and beliefs of self-help strategies; (iv) knowledge and beliefs about professional help available; (v) attitudes that facilitate recognition and appropriate help-seeking behaviors; and (vi) knowledge of how to seek mental health information. Studies of the general population revealed that depression and limited depression literacy often co-exist (Francis et al., 2007), and individuals with limited depression literacy were almost three times as likely as those with sufficient depression literacy to undergo depressive symptoms (Gazmararian et al., 2000). Moreover, documented evidence suggests that low levels of PoDLi may contribute to new mothers’ minimization and normalization of their symptoms of PPD (Bilszta et al., 2010; Callister et al., 2019), and lead them to engage in maladaptive behaviors such as alcohol consumption (Guy et al., 2014). On the contrary, if a person’s depression literacy is promoted, he/she will respond better to depression treatments and be more willing to seek professional help for depression symptoms (Francis et al., 2007). Given the close relationship between depression literacy and the occurrence, recognition, treatment, and management of depression (Gazmararian et al., 2000; Francis et al., 2007), accurately assessing the PoDLi level among perinatal women is critical, which is a prerequisite to implement PPD awareness improving programs for early recognition and treatment on PPD.

Nevertheless, only two studies have been carried out to investigate the depression literacy level in perinatal women by using the 22-item depression literacy questionnaire (Fonseca et al., 2017) and vignettes for major depressive episodes (Buist et al., 2007) that are both designed for the general public and may lead to deviations in evaluation results. Given the above situation, Mirsalimi et al. (2020) initially developed the postpartum depression literacy scale (PoDLiS) in the guide of Jorm’s MHL framework for measuring perinatal women’s PoDLi levels. To date, PoDLiS has been adapted into the Malay version (Hairol et al., 2021), applied in Iran (Mirsalimi et al., 2020), India (Poreddi et al., 2021), and Malaysia (Hairol et al., 2021), and tested to be reliable and valid. But it is unclear whether PoDLiS is generalizable to other cultural contexts, as the existence and adaptation of one scale version does not guarantee measurement equivalence across other populations (Canino and Alegría, 2008; Onchonga et al., 2021).

As the most populous country in the world, China accounts for nearly one-fifth of the world’s population. According to the research results from different provinces, the PPD incidence in China ranged from 15 to 20% and still showed a significant increasing trend (Mu et al., 2019). Specifically, as per the most recent pregnancy and birth records (National Bureau of Statistics of China, 2018), it translates to potentially 5–7 million women with PPD—the largest numbers for any single country. Worse still, in China, there is a serious shortage of mental health professionals, insufficient promotion of related knowledge and maternal psychological monitoring, as well as an unoptimistic situation for PPD recognition and treatment (Xiang et al., 2018). Accordingly, it is imperative to extensively evaluate the PoDLi level in Chinese perinatal women, which is the first step in developing a PoDLi improvement program to ameliorate the prevention, recognition, and treatment of PPD. However, as far as we know, most of the studies in China focused on the prevalence of PPD (Mu et al., 2019; Nisar et al., 2020), while no study has investigated Chinese perinatal women’s PoDLi level. The lack of condition-specific measures for PoDLi may count a large part of the reason for this phenomenon. Thus, there is an increasing demand to develop a valid and reliable instrument for PoDLi assessment in China. Due to the high cost and long period required for developing a new scale, translating and testing a well-developed existing scale from another country seems to be an informed choice (Li et al., 2016). Thence, this study aimed to (World Health Organization [WHO], 2016) translate and cross-culturally adapt the PoDLiS to the Chinese context and (World Health Organization [WHO], 2013) psychometrically validate the Chinese version of PoDLiS (C-PoDLiS).

## Materials and methods

### Study design and participants

**FIGURE 1:**
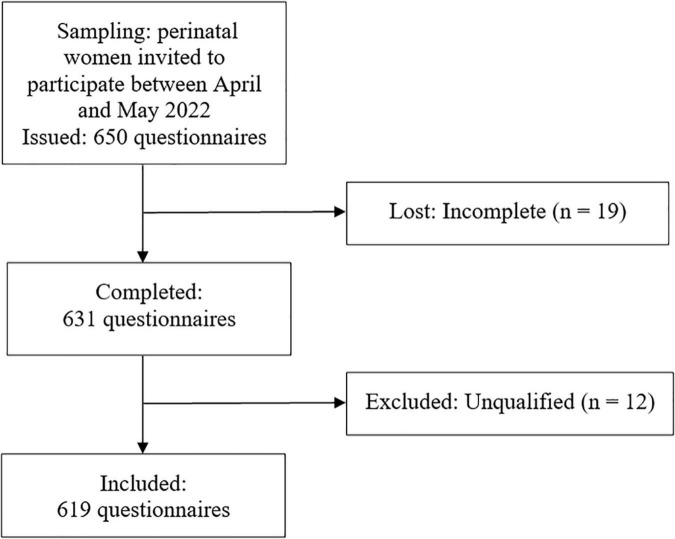
Flowchart of the data collection.

A cross-sectional survey study was performed for the translation, cross-cultural adaptation, and psychometric property validation of the PoDLiS in Mainland China. The Strengthening the Reporting of Observational Studies in Epidemiology statement (von Elm et al., 2007) was used to report the findings. Perinatal women from the maternity wards of a tertiary hospital in Hangzhou, Zhejiang Province, were recruited *via* the convenience sample method from April to May 2022. Self-administered pen-and-paper questionnaires were distributed by two authors (both are well-trained nursing Ph.D. candidates) and were retrieved immediately after being completed to check the missing content and ensure the questionnaire’s integrity. Per the methodological recommendations, 10 cases per candidate item are required for exploratory factor analysis (EFA), and the sample size should be at least 200 to be able to build a confirmatory factor analysis (CFA) model (Wu, 2010). Thus, a minimum sample size of 510 was estimated “*a priori*.” A total of 650 questionnaires were finally distributed due to the sufficient sample sources. After eliminating invalid questionnaires, 619 questionnaires were suitable for statistical analysis (Figure 1). The sample was splitted into two parts according to the order of questionnaire collection to perform EFA (sample 1: *N* = 310) and CFA (sample 2: *N* = 309).

The following criteria were employed for inclusion, which were similar to those of Mirsalimi et al. (2020) that tested psychometric characteristics of the PoDLiS: (a) being at least 18 years old; (b) being pregnant or within 12 months of giving birth; (c) being able to read and articulate in Chinese (primary education must be completed); and (d) agreed to participate in this study. Perinatal women with serious medical illness requiring individualized assessment, tracking as “a high-risk pregnancy” (e.g., diabetes, hypertension), with positive screening for psychosis, suicidality, and substance abuse disorders, or with communication barriers (deafness or blindness) were excluded.

### Instruments

#### Demographic characteristic

A self-compiled questionnaire comprising 11 questions was applied to collect socio-demographic information from participants, including age, education level, religion, monthly family income, residence, employment status, marital status, parity, perinatal period, whether they had suffered from depression or other mood disorders previously, and whether they had studied psychology before.

#### Postpartum depression literacy scale

The PoDLiS is a self-reported instrument designed to assess the level of PoDLi in perinatal women. The original PoDLiS was constructed by Mirsalimi et al. (2020) and is composed of 31 items (8 reverse-scored items) and 7 dimensions: ability to recognize PPD; knowledge of risk factors and causes; knowledge and belief of self-care activities; knowledge about professional help available; beliefs about professional help available; attitudes that facilitate recognition of PPD and appropriate help-seeking; and knowledge of how to seek information related to PPD. Each item of PoDLiS is valued on a 5-point Likert scale from 1 (strongly disagree/not likely at all) to 5 (strongly agree/very likely). The total score for PoDLiS as well as scores for each dimension are calculated by adding up the coded values and then dividing them into the number of items, ranging from 1 to 5, with higher scores denoting better PoDLi level. The original version of PoDLiS has good construct validity and internal reliability. The Cronbach’s α for the whole scale was 0.78, and the α coefficients for seven domains were between 0.70 and 0.83.

#### Mental health literacy scale

The mental health literacy scale (MHLS) was developed by O’Connor and Casey (2015) to assess the level of MHL. The Chinese version of MHLS includes 35 items and 6 subscales (the capacity to recognize mental health disorders; knowledge of risk factors; familiarity with self-treatment; awareness of available professional help; knowledge of how to seek information; and attitudes that promote recognition or effective aid-seeking behavior), of which the 1–15 items have a 4-point Likert scale (1–4) and the 16–20 items have a 5-point Likert scale (1–5) (Ma, 2019). The total score of MHLS ranged from 35 to 160, with higher scores representing an adequate MHL. As reported, the Chinese version of MHLS has good reliability and validity and can be applicable to different populations in China (Han et al., 2019; Ma, 2019; Chen et al., 2021). The Cronbach’s α of MHLS was 0.840 for this study. The MHLS was completed by a sample of 290 people for testing concurrent validity.

#### Attitudes toward seeking professional psychological help short form scale

The attitudes toward seeking professional psychological help short form scale (ATSPPH-SF) was developed by Fischer and Farina (1995). The Chinese version of ATSPPH-SF consists of 10 items and 2 domains: openness to seeking treatment for emotional problems and value and need in seeking treatment (Fang et al., 2019). Item responses are based on a 4-point Likert scale (0–3). The total score of ATTSPH-SF ranges from 0 to 30, with higher scores indicating a higher propensity to seeking help. The reliability and validity of the Chinese version of the ATSPPH-SF has been extensively verified (Fang et al., 2019; Chen et al., 2020). In the current study, a Cronbach’s α of 0.766 was obtained. The ATSPPH-SF was completed by a sample of 100 people to test predictive validity.

### Study procedure-phase I: Instrument development

Permission to translate the English version of PoDLiS to the Chinese language was granted by the developers (Mirsalimi, PhD) (Mirsalimi et al., 2020). An internationally accepted multiphase translation guideline (Beaton et al., 2000) was followed in the translation and validation process.

#### Forward translation

Two fluent translators in both languages (one was an obstetrics nursing postgraduate who was familiar with the concepts explored, while the other didn’t have a medical background) independently translated the original PoDLiS into Chinese.

#### Synthesis of the two translated versions

After any discrepancies about the words, phrases, and items in the translation process were fully discussed by two translators, a synthesis forward translation version was obtained.

#### Back translation

Two bilingual native English speakers, who were blinded to the original PoDLiS with no medical background, translated the synthesis version back into English independently, creating two backward translation versions.

#### Specialist committee review

All reports were compared and reviewed by a specialist committee that was composed of eight experts (an obstetrics expert, a psychologist, a methodologist, a linguistics professional, and four translators) to reach a consensus on discrepancies and achieve semantic, idiomatic, cultural, and conceptual equivalence. Additionally, the expert panel referred back to the original English version to capture the accurate meaning of each item. Ultimately, we sent the synthesis forward translation version, two back-translation versions, and unaddressed translation issues to the developer for confirmation, then integrated their feedback to generate an integrated version. All problems, discrepancies, and discussion in each translation stage were documented.

#### Evaluation of content validity

Expert consulting was carried out to assess the content validity of the C-PoDLiS and to confirm whether the items were designed properly to create the constructs. The survey consisted of four parts of content, including biographical information (education level, working experience, etc.), the original English PoDLiS, the integrated version of C-PoDLiS, and an assessment file for content equivalence (using a 4-point ordinal rating scale: 1 = irrelevant; 2 = somewhat relevant and needs major modification; 3 = quite relevant but needs minor modification; 4 = extremely relevant). Experts were also asked regarding the clarity of each item and their revision suggestions. The survey was emailed to six experts in accordance with the recommendation of guideline (Beaton et al., 2000) (three nursing professors, two clinical experts in obstetric care, and a mental health expert) and got feedback from all of them. Then, the specialist committee discussed and integrated all revision suggestions to generate a pre-final version for pilot testing.

#### Pilot testing

According to the guideline that recommends a sample size of 30–40 participants for pre-testing (Beaton et al., 2000), the pre-final version was administered in the same tertiary hospital to a purposive sample of 33 eligible perinatal women with diverse characteristics (in terms of age, education, and occupational background) to examine face validity (comprehension of the meaning and appropriateness of the wording of test items), simplify the item wording, and assess the time consumption. The 33 samples were not included in the psychometric validation. Since no major suggestions emerged, the preliminary C-PoDLiS was only fine-tuned compared to the pre-final version.

### Study procedure-phase II: Instrument validation

According to the recommendation in the COSMIN (Consensus-based Standards for the Selection of Health Status Measurement Instruments) checklist (Mokkink et al., 2010a; Prinsen et al., 2018), the psychometric properties of the C-PoDLiS were assessed in terms of item analysis, structural validity, convergent and discriminant validity, reliability, a floor/ceiling effect, and criterion validity.

### Data analysis

Data were analyzed using SPSS V.25.0, AMOS V.23.0, and jamovi V.2.2.5. Enumeration data was described by frequency and percentage (%), and the measurement data was described by mean and standard deviation (SD). *P* values < 0.05 were considered statistically significant.

#### Content validity

The content validity index (CVI) was computed at both the item level (I-CVI) and the scale level (S-CVI). The I-CVI is the proportion of content experts giving either 3 or 4 on a 4-point scale, whereas the S-CVI is the average of the total I-CVI on the scale. As suggested by Polit and Beck (2009), I-CVI ≥ 0.78 and S-CVI ≥ 0.80 are considered to be appropriate if the number of experts is ≥6.

#### Item analysis

Item analysis was performed using the following analyses: (1) extreme groups’ analysis: items should be significantly discriminated between the upper 27% and the lower 27% of scoring groups (Odukoya et al., 2018); (2) critical ratio (CR): items with CR < 3.0 and a *P* value > 0.05 were deleted (Wu, 2010); (3) correlation coefficient method: an item was retained when its score was significantly correlated with the total scale score or the item-total correlation value was 0.30–0.80 (Wu, 2010); (4) Cronbach’s α or McDonald’s ω greatly improved (increasing the value of the α or ω coefficient for the overall scale by 0.5 or more) if an item was deleted (Wu, 2010).

#### Structure validity

To establish the structural validity of the C-PoDLiS, EFA was first conducted, and then the results were verified *via* CFA. Prior to EFA, the Kaiser–Meyer–Olkin (KMO) test (≥0.6 is considered acceptable) and Bartlett’s test of sphericity (significant tests demonstrate adequacy) were conducted to ensure the data’s suitability for factor analysis (Hu et al., 2019). For EFA analyses, the principal component analysis with the oblique rotation method (promax criterion) was applied as the correlation between the factors was >0.3 (Wu, 2010; Hu et al., 2019). The criterion for factor extraction and item retention was eigenvalues > 1.0, factor loadings > 0.45 (Wu, 2010), and items with consistent predefined sub-dimensions. The scree plot was also examined to determine the factor structure. For CFA analyses, the maximum likelihood estimation method was used to estimate the parameters. The *t*-value and factor loading of each item, as well as the goodness-of-fit, were considered when commenting on the fit of the CFA model. Items with either a *t*-value < 1.96 (Wu, 2010) or a factor loading < 0.32 (Qu et al., 2021) should be deleted. The goodness-of-fit of the CFA model was measured by the following indices: χ^2^/*df*, comparative fit index (CFI), goodness of fit (GFI), Tucker–Lewis index (TLI), incremental fit index (IFI), and root mean square error of approximation (RMSEA) (Brown, 2006). As recommended, a CFA model is adequately fitted when 1 < χ^2^/*df* < 3, RMSEA < 0.08, whereas CFI, GFI, TLI, and IFI > 0.90 (Brown, 2006).

#### Convergent and discriminant validity

Average variance extracted (AVE) was used to assess the internal convergent validity of a factor, with a score ≥ 0.5 indicating satisfactory convergent validity (Fornell and Larcker, 1981). To show discriminant validity, the √AVE score should exceed each of its correlations with other factors (Fornell and Larcker, 1981; Hair et al., 2010).

#### Reliability

Scale reliability was evaluated in terms of internal consistency and stability. Internal consistency was tested by calculating the Cronbach’s α and McDonald’s ω (Peter, 2014). The intraclass correlation coefficient (ICC) was calculated *via* the test-retest reliability method to evaluate the stability of the C-PoDLiS (Perinetti, 2018). Thirty-one perinatal women who were randomly selected from the total samples completed the preliminary C-PoDLiS at a 2-week interval to establish test-retest reliability. Sufficient reliability is based on values of Cronbach’s α, McDonald’s ω, and ICC equal to or greater than 0.70 (Hair et al., 2010), whereas scores of these indicators between 0.60 and 0.70 signifie an acceptable level of reliability (Wu, 2010).

#### Floor/ceiling effect

The floor/ceiling effect (the percentage of people scoring at the bottom and top of a scale) for the total scale were analyzed to assess the interpretability. Less than 15% of responses with the lowest or highest score were deemed acceptable, defining no substantial floor and ceiling effects (Mokkink et al., 2010b).

#### Criterion validity

To evaluate the concurrent validity and predictive validity, the Spearman rank correlations were analyzed between the C-PoDLiS and other instruments (MHLS and ATSPPH-SF). The correlation of |*r*| = 0.10–0.30, |*r*| = 0.31–0.60, and |*r*| = 0.61–1.00 were considered low, moderate, and high, respectively (Andresen, 2000). According to previous studies, we hypothesized that the C-PoDLiS score would be positively associated with both the MHLS score and the ATSPPH-SF score.

### Ethics consideration

This study was reviewed and approved by the hospital’s Research Ethics Review Committee (IRB-20220190-R). All the procedures that were carried out in this study were consistent with the 1964 Declaration of Helsinki and its later amendments (World Medical Association [WMA], 2008). The participants were informed of the purpose of the study as well as the anonymity and confidentiality of the survey before commencement. They were also informed that they can withdraw at any stage while being surveyed without any penalty. Written informed consent was obtained from all participants and they agreed that their data could be used in the project. The participants were not financially compensated. The survey did not disclose any personal information.

## Results

### Cross-cultural adaptation, face validity, and content validity

Based on the results of expert consultations, evidence from literature, and feedback from the pilot testing, some adjustments and modifications were made to obtain the preliminary version of the C-PoDLiS (see Supplementary Table 1). First, 2 new items were added (item 15 and item 21). Second, the items “Treatment for PPD, provided by a mental health professional, can be effective” and “Psychotherapy (for example, talking therapy or counseling) can be effective in treating PPD” of the original scale were merged into item 18; the items “I know where to seek information about PPD” and “I know how to use various sources to seek information about PPD” of the original scale were merged into item 28. Third, some words and phrases were changed. For instance, “mental health professional” in item 5 of the original scale was adjusted to “psychotherapist or psychiatrist.” Given that only one item was left in the dimension of “Knowledge about professional help available” after the item mergence, this dimension was integrated with the dimension of “Beliefs about professional help available,” generating a new dimension named “Knowledge and beliefs about professional help available.” In the pilot testing, 33 participants stated that the wording of the C-PoDLiS was clear and they had little difficulty understanding it. In the second round of expert consultations, the S-CVI was 0.920, and the I-CVI ranged from 0.833 to 1.00. Eventually, the preliminary C-PoDLiS with 31 items and 6 factors was generated for psychometric evaluation.

### Sample characteristics

A total of 619 (out of a possible 650) perinatal women completed the survey (Figure 1), for an effective response rate of 95.23%. The mean age of participants was between 20 and 42 years (mean = 30.85, SD = 4.03). Perinatal women who were non-religious, married, lived in urban areas, had a specialty/bachelor’s degree, in the postpartum period, and were primiparous counted the most. The detailed demographics of participants are shown in Table 1.

**TABLE 1:** Characteristics of the included participants.

Characteristics	Mean (standard deviation)/*N* (%)		
	
	Total sample *N* = 619	Sample 1 *N* = 310	Sample 2 *N* = 309
**Age, years**	30.85 (4.03)	30.84 (3.95)	30.87 (4.11)
**Education**			
Junior school or below	30 (4.9)	15 (4.9)	15 (4.9)
High school/Specialized secondary school	50 (8.1)	28 (9.0)	22 (7.1)
Specialty/Bachelor	444 (71.7)	222 (71.6)	222 (71.8)
Postgraduate or above	95 (15.3)	45 (14.5)	50 (16.2)
**Religion**			
Yes	82 (13.2)	44 (14.2)	38 (12.3)
No	537 (86.8)	266 (85.8)	271 (87.7)
**Marital status**			
Married	609 (98.4)	305 (98.4)	304 (98.4)
Unmarried	6 (1.0)	2 (0.6)	4 (1.3)
Divorced	4 (0.6)	3 (1.0)	1 (0.3)
**Residence**			
Rural	131 (21.2)	70 (22.6)	61 (19.7)
Urban	488 (78.8)	240 (77.4)	248 (80.3)
**Household monthly income (Chinese Yuan)**			
≤10000	139 (22.5)	73 (23.6)	66 (21.4)
10001–20000	267 (43.1)	126 (40.6)	141 (45.6)
>20000	213 (34.4)	111 (35.8)	102 (33.0)
**Employment status**			
Employed	562 (90.8)	279 (90.0)	283 (91.6)
Unemployed	57 (9.2)	31 (10.0)	26 (8.4)
**Perinatal period**			
Pregnancy	365 (59.0)	196 (63.2)	169 (54.7)
Postpartum	254 (41.0)	114 (36.8)	140 (45.3)
**Parity**			
Primiparity	435 (70.3)	213 (68.7)	222 (71.8)
Multiparity	184 (29.7)	97 (31.3)	87 (28.2)
**Whether suffered from depression or other mood disorders before**			
Yes	46 (7.4)	23 (7.4)	23 (7.4)
No	573 (92.6)	287 (92.6)	286 (92.6)
**Have you ever learned psychology?**			
Yes	203 (32.8)	93 (30.0)	110 (35.6)
No	416 (67.2)	217 (70.0)	199 (64.4)

### Psychometric analysis

#### Item analysis

After deleting two items (19 and 20) with no significant difference in the extreme group comparison, the CR values of the remaining 29 items were >3.0. Then, according to the results of the correlation coefficient method, all items met the standard and were retained. Specifically, the score of item 24 was significantly correlated with the total scale score (*P* < 0.01) despite the coefficient value being 0.236 (<0.3), whereas the item-total correlation values ranged from 0.303 to 0.593 for the remaining 28 items (Table 2). No individual item was found to greatly increase the Cronbach’s α or McDonald’s ω if deleted.

**TABLE 2:** Item analysis.

Item	Extreme group comparison	Item-total correlation	Cronbach’s α if item deleted	McDonald’s omega if the item is deleted	Numbers of substandard indicators	Note
	**Criterial ratio**					
1	9.688**	0.553**	0.844	0.855	0	Retained
2	8.258**	0.446**	0.846	0.857	0	Retained
3	8.937**	0.465**	0.846	0.857	0	Retained
4	10.669**	0.593**	0.844	0.854	0	Retained
5	8.711**	0.561**	0.844	0.855	0	Retained
6	7.560**	0.448**	0.846	0.856	0	Retained
7	7.819**	0.440**	0.848	0.858	0	Retained
8	9.429**	0.531**	0.844	0.855	0	Retained
9	8.323**	0.524**	0.844	0.855	0	Retained
10	10.54**	0.545**	0.843	0.854	0	Retained
11	9.002**	0.513**	0.844	0.855	0	Retained
12	7.651**	0.494**	0.844	0.854	0	Retained
13	6.744**	0.445**	0.846	0.856	0	Retained
14	5.166**	0.331**	0.851	0.860	0	Retained
15	7.883**	0.505**	0.844	0.853	0	Retained
16	7.777**	0.481**	0.845	0.855	0	Retained
17	8.594**	0.551**	0.844	0.852	0	Retained
18	6.565**	0.385**	0.848	0.858	0	Retained
19	**−0.201**	**0.002**	0.859	0.868	2	Deleted
20	**1.683**	0.127*	0.856	0.865	1	Deleted
21	5.281**	0.303**	0.848	0.858	0	Retained
22	9.013**	0.501**	0.844	0.856	0	Retained
23	6.937**	0.430**	0.848	0.858	0	Retained
24	4.239**	0.236**	0.853	0.863	0	Retained
25	7.025**	0.418**	0.848	0.859	0	Retained
26	8.179**	0.458**	0.848	0.859	0	Retained
27	7.657**	0.456**	0.848	0.859	0	Retained
28	6.454**	0.410**	0.848	0.858	0	Retained
29	5.462**	0.378**	0.847	0.857	0	Retained
30	5.365**	0.361**	0.848	0.858	0	Retained
31	5.760**	0.364**	0.847	0.858	0	Retained

C-PoDLiS, Chinese version of the postpartum depression literacy scale.

*P < 0.05; **p < 0.01.

Bold values are characters that mean the indicator is substandard.

The Cronbach’s α of the C-PoDLiS was 0.851; The McDonald’s omega coefficient of the C-PoDLiS was 0.869.

#### Structure validity

**FIGURE 2:**
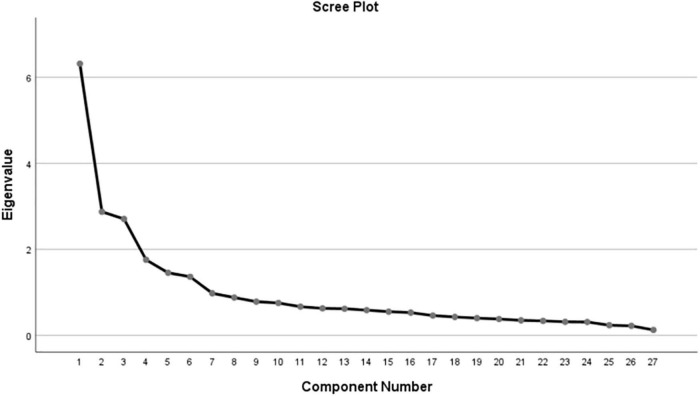
The scree plot.

The results of 29 items showed a KMO value of 0.821 and the Bartlett spherical test value of 3490.603 (χ^2^ = 3490.603, *df* = 351, *p* < 0.001), which demonstrated that the data set was adequate for EFA. In the first EFA, the item 7 and item 14 were removed sequentially because they were inconsistent with the pre-defined subscales. Ultimately, on the basis of the scree plot (Figure 2) and a second EFA, six factors were extracted with eigenvalues > 1.0 (6.32, 2.88, 2.71, 1.76, 1.45, and 1.36) that explained 61.00% of the total variance, of which factors 1, 2, 3, 4, 5, and 6 explained 23.39, 10.65, 10.03, 6.51, 5.39, and 5.04% of the variance, respectively (Supplementary Table 2). Moreover, the factor loadings were >0.45, and the remaining 27 items loaded onto the predefined factor.

**FIGURE 3:**
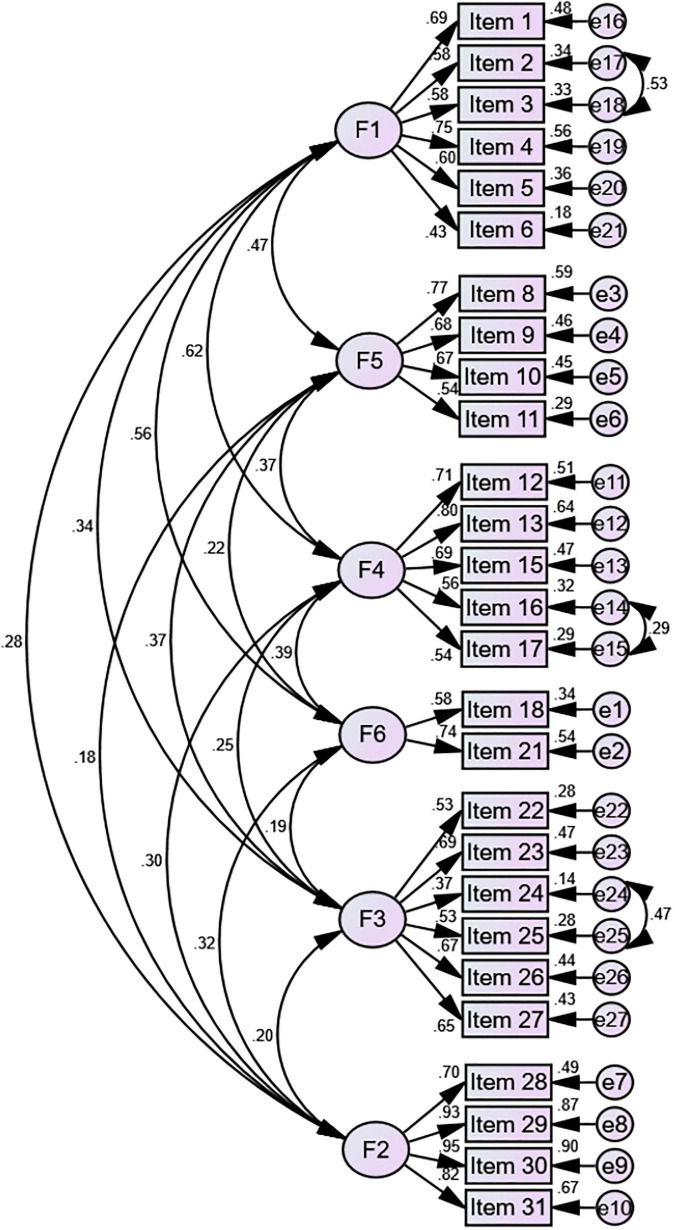
Modified confirmatory factor analysis of the six factor 27-item model.

Confirmatory factor analysis was conducted to confirm the EFA-derived 27-item six-factor structure. The *t*-values of all items were higher than 1.96 (the results were not shown specifically), and all items presented factor loadings > 0.32 (Figure 3). The initial model indices suggested an inadequate fit on the basis of GFI, IFI, CFI, and TLI (χ^2^/*df* = 2.062, RMSEA = 0.059, GFI = 0.863, IFI = 0.897, CFI = 0.895, and TLI = 0.881). Three paths of covariance between errors were added based on the modification indices. The adjusted model achieved a good fit to the data (χ^2^/*df* = 1.532, RMSEA = 0.042, GFI = 0.900, IFI = 0.949, CFI = 0.948, and TLI = 0.940) (Figure 3).

#### Convergent and discriminant validity

The values of AVE for four subscales were above the desired threshold of 0.50 and for the other two subscales were very close to 0.5. Moreover, all subscales’ √AVE scores exceeded each of their correlations with other subscales (Table 3), indicating good discriminant validity.

**TABLE 3:** Correlation coefficient and AVE.

C-PoDLiS	AVE	Inter-dimension correlations					
		
		1	2	3	4	5	6
Dimension 1	0.495	**0.704**					
Dimension 2	0.722	0.104	**0.850**				
Dimension 3	0.486	0.175**	0.150**	**0.697**			
Dimension 4	0.557	0.463**	0.219**	0.233**	**0.746**		
Dimension 5	0.620	0.474**	0.165**	0.327**	0.377**	**0.787**	
Dimension 6	0.664	0.252**	0.157**	0.120*	0.239**	0.190**	**0.815**

AVE, average variance extracted; C-PoDLiS, Chinese version of the postpartum depression literacy scale.

**P < 0.01; *P < 0.05.

Bold characters show the square of root of average variance extracted values.

#### Reliability

The Cronbach’s α of the C-PoDLiS was 0.862, and the subscales ranged from 0.679 to 0.880. The McDonald’s ω was 0.869 for the total scale, whereas the subscales ranged from 0.689 to 0.889. And the ICC for the total scale was 0.856 and for the six factors ranged from 0.633 to 0.748 (Table 4).

**TABLE 4:** Reliability analysis of the C-PoDLiS.

	C-PoDLiS	Dimension 1	Dimension 2	Dimension 3	Dimension 4	Dimension 5	Dimension 6
Cronbach’s α coefficient	0.862	0.82	0.88	0.788	0.817	0.797	0.679
McDonald’s omega coefficient	0.869	0.823	0.889	0.79	0.826	0.799	0.689
Intraclass correlation coefficient	0.856	0.721	0.721	0.633	0.671	0.699	0.748

C-PoDLiS, Chinese version of the postpartum depression literacy scale.

#### Floor/ceiling effect

The C-PoDLiS scores ranged from 1 to 5. These results revealed no floor and ceiling effects as no perinatal women recorded the highest or lowest possible score.

#### Criterion validity

Regarding concurrent validity, the C-PoDLiS demonstrated significant positive correlations with MHLS and its six dimensions among 290 perinatal women (*r* = 0.155–0.581, *p* < 0.01) (Table 5). For predictive validity, the C-PoDLiS also had significant positive correlations with ATSPPH-SF and its two dimensions in a cross-sectional study of 100 perinatal women (*r* = 0.361–0.432, *p* < 0.01) (Table 6).

**TABLE 5:** Correlations of the C-PoDLiS and MHLS subscales.

	MHLS	Dimension 1	Dimension 2	Dimension 3	Dimension 4	Dimension 5	Dimension 6
C-PoDLiS	0.581**	0.524**	0.399**	0.212**	0.329**	0.155**	0.391**

C-PoDLiS, Chinese version of the postpartum depression literacy scale; MHLS, mental health literacy scale.

**P < 0.01.

**TABLE 6:** Correlations of the C-PoDLiS and ATSPPH-SF subscales.

	ATSPPH-SF	Dimension 1	Dimension 2
C-PoDLiS	0.432**	0.361**	0.397**

C-PoDLiS, Chinese version of the postpartum depression literacy scale; ATSPPH-SF, attitudes toward seeking professional psychological help short form scale.

**P < 0.01.

### Scale summary

The final version of the C-PoDLiS has 27 items grouped into six domains. The detailed contents and the instructions for scoring are shown in the Supplementary Table 1. Regarding the PoDLi of participants in this study, the total mean score was 3.80 ± 0.38. The average scores of each factor ranking from the highest to the lowest were 4.25 ± 0.64 (factor 5: knowledge of risk factors and causes), 4.16 ± 0.51 (factor 4: knowledge and belief of self-care activities), 3.81 ± 0.60 (factor 3: attitudes toward promoting recognition of PPD or appropriate help-seeking behaviors), 3.65 ± 0.61 (factor 1: recognition abilities of PPD), 3.45 ± 0.71 (factor 6: knowledge and beliefs about professional help available), and 3.30 ± 0.70 (factor 2: ability to seek and judge information related to PPD).

## Discussion

The PoDLi is closely associated with the occurrence, recognition, and treatment of PPD (Gazmararian et al., 2000; Francis et al., 2007). Consequently, it is essential to find an evidence-based, convenient, and practical instrument specifically to quantity the level of PoDLi among perinatal women in China. To our knowledge, the present study is the first to cross-culturally adapt and validate the PoDLiS among the Chinese population. The psychometric validation was conducted under the guidelines of the COSMIN checklist (Mokkink et al., 2010a; Prinsen et al., 2018). The results suggested satisfactory internal consistency (Cronbach’s α = 0.862 and McDonald’s ω = 0.869), adequate stability (ICC = 0.856), sufficient or acceptable validity (content validity, structural validity, convergent and discriminant validity, concurrent validity, and predictive validity), and no floor or ceiling effect of the C-PoDLiS. The six-factor model explained 61.00% of the total variance. The participants completed the scale in a maximum of 10 min. Overall, the C-PoDLiS can serve as a valid, reliable, and practicable measurement tool for evaluating the level of PoDLi among perinatal women in mainland China.

Results of this study painted a preliminary picture of the level of PoDLi in perinatal women in China. The findings suggested that the PoDLi level (3.80 ± 0.38) among Chinese perinatal women was in the moderate range, which was very close to the findings in the Iran (3.79 ± 0.39) (Mirsalimi et al., 2020) and Malaysia (3.78 ± 0.37) (Hairol et al., 2021) samples, and a study in India (Poreddi et al., 2021) showed that only half of postpartum mothers had adequate levels of PoDLi. Consequently, there is an imperative need, both in China and many other developing countries, to sensitize perinatal women about PPD *via* various approaches such as presenting knowledge of PPD in community campaigns and pregnancy schools, thereby creating awareness of PPD and improving help-seeking behaviors. Moreover, the lowest mean score of factor was observed on factor 2: ability to seek and judge information related to PPD (3.30 ± 0.70) in this study, which was congruent with the study in India (Poreddi et al., 2021) but different from the study in Iran (Mirsalimi et al., 2020). Even more interesting is that the lowest-scoring dimension in the study from Iran (beliefs about professional help available) (Mirsalimi et al., 2020) was precisely the highest-scoring dimension in India (Poreddi et al., 2021). Accordingly, it could be concluded that the specific gaps and deficient elements in PoDLi vary widely among perinatal women in different countries, so PoDLi improvement programs in the future should be culturally tailored.

In the process of translation and cross-cultural adaptation, we strictly followed the international multiphase translation guidelines (Beaton et al., 2000) and sought advice from the developer of PoDLiS (Dr. Mirsalimi) to guarantee the content equivalence between the source and target versions. Compared with the PoDLiS, four items were merged, respectively, into two, two new items were added, and minor modifications to some words and statements were made in the preliminary C-PoDLiS, which has reduced the redundancy, improved the succinctness, and guaranteed the comprehensiveness of the scale’s content, as well as made the C-PoDLiS more in line with China’s actual condition and Chinese expression habits. What deserves to be mentioned is that most of the items had cultural equivalent terms in Chinese, so the translation could be completed without extensive cultural adaptation. In addition, based on the principle that a single item cannot be a dimension by itself (Wu, 2010), two dimensions of the original PoDLiS were merged. Therefore, the preliminary C-PoDLiS consisted of 6 domains, which was more in accordance with Jorm’s definition of MHL (Jorm, 2000) in spite of a slight difference from the 7-domain structure of the original PoDLiS (Mirsalimi et al., 2020). Eventually, the 33-participant pilot testing confirmed the good face validity of the C-PoDLiS and the second round of expert consultations revealed excellent content validity (I-CVI = 0.86–1.00 and S-CVI = 0.915).

With regard to item analysis, two items (item 13: “Antidepressants are addictive” and item 14: “Antidepressants cause brain damage”), both in the dimension of “Knowledge and beliefs about professional help available,” were omitted because of the non-significant differences in the extreme group comparison. One possible reason for the non-significant differences may be a general lack of knowledge of antidepressants by perinatal women (Muzik and Hamilton, 2016), which made it difficult to score these items correctly even for participants in the group with a higher score of PoDLiS. This phenomenon reinforces the notion that healthcare professionals are necessary to be proactive in improving perinatal women’s knowledge about professional treatment of PPD. Noteworthily, the new added item (item 33: “Antidepressants are effective in treating PPD”) that has similar content (all were about the efficacy of antidepressants) but a reverse scoring compared to the two deleted items, was finally retained in C-PoDLiS, which could remedy the defect of the damaged content comprehensiveness of the scale to some extent caused by the above-mentioned items’ deletion.

Factor analysis was carried out to determine the degree to which participants’ scores on C-PoDLiS were an adequate reflection of the dimensionality of the structure being measured and the conceptual framework on which it is based. In EFA, two items (item 14: “Religious practices, prayer and going to holy shrine are helpful for the prevention and management of PPD,” and item 7: “How likely is it that PPD might be caused by a genetic or inherited problem”) were eliminated because they loaded on a different factor compared to their pre-defined factor, which could not be explained theoretically. The reason for the mismatch of item 14 may be resulted from the difference in sample characteristics. To be specific, 86.75% (537/619) of the participants recruited in this study were non-religious, so religious activities could hardly be considered as self-care activities for most of them, while the data set in the original PoDLiS was from Iranian perinatal women who believe in Islam with great probability (Mirsalimi et al., 2020). What should be pointed out is that in some areas of mainland China, especially in the settlements and autonomous regions of ethnic minorities, religion is relatively common. Hence, considering that item 14 may be meaningful to some perinatal crowds in China, it is necessary for future studies to apply appropriate adjustments and retest this item. For item 7, whether or not genetic and inherited problems are the etiology of PPD is still controversial at present (Starnawska et al., 2021; Kiewa et al., 2022). Meanwhile, some experts pointed out in the first round of expert consultations that since genetic and inherited problems are incurable, item 7 could be a double-edged sword for perinatal women; on one hand, this item could enlighten the participants with genetic and inherited problems related to depression to pay more attention to their mental health during the perinatal period, while on the other hand, it might increase their concerns about developing PPD. After due consideration of experts’ suggestions and the results of EFA, item 7 was finally removed. Ultimately, the 27-item 6-factor model that was generated from EFA was in line with the pre-designed framework in the preliminary C-PoDLiS and explained a much higher proportion of the total variance than the original 31-item PoDLiS (61.00% vs. 49.00%), which reflected that the C-PoDLiS was more in accord with the Chinese cultural background and medical environment after adjustment. In CFA, the performance of the initial model fit indices was less satisfactory, so based on the modification recommendations, three error covariances were added (Figure 3). Before the modification, we had checked the specific contents of relevant items and believed that these covariances were plausible as there were indeed strong correlations between these items. Ultimately, all of the goodness-of-fit indexes in the adjusted model reached a good level. In addition, acceptable convergent validity (AVE = 0.486–0.722) and good discriminant validity were also given the reasonable fit of the 6-factor model. Generally speaking, C-PoDLiS is in line with the definition of PoDLi and is adequate to measure Chinese perinatal women’s level of PoDLi.

For reliability, the total Cronbach’s α was estimated to be 0.862 for excellent internal consistency, indicating that all items contribute to the global construct measured, which is far higher than the original version (Cronbach’s α 0.78) (Mirsalimi et al., 2020) and the Malay version (Cronbach’s α 0.73) (Hairol et al., 2021). When considering the factor structure, α values exceeding 0.7 were discovered for five factors, whereas factor 4 consisting of 2 items had a lower but acceptable Cronbach’s α (0.679). As Cronbach’s α is very sensitive to the number of items in scales, it is common to detect lower α values in factors with a few items (Streiner, 2003). The results of McDonald’s ω (0.689–0.0.889), a more accurate coefficient of internal consistency, further supported the satisfactory reliability of the C-PoDLiS. The ICC scores of the PoDLiS (0.633–0.856) were assessed for the first time in this study and revealed appropriate stability for the scale. This feature is quite important when the C-PoDLiS is applied to evaluate the effectiveness of interventions on ameliorating the PoDLi level. Additionally, the absence of floor/ceiling effects for the total score of C-PoDLiS suggested that C-PoDLiS could discriminate between participants at either extreme of the scale and confirmed its applicability in Chinese perinatal women.

The evaluation of criterion validity of C-PoDLiS represented a novelty because it had never been assessed before. In terms of concurrent validity, since there is no adequate instrument as a gold standard for assessing MHL or depression literacy, the concurrent validity of C-PoDLiS was measured with MHLS that has been extensively tested for good reliability and validity and has been widely applied among various Chinese populations (Han et al., 2019; Ma, 2019; Chen et al., 2021). As expected, statistically significant correlations between the scores of the C-PoDLiS and the MHLS were discovered (Table 6), suggesting that the C-PoDLiS was sensitive enough to assess similar features as the MHLS. However, all of the association coefficients were moderate or small, which might be caused by the differences in essential attributes of the items between the two scales, as the MHLS measures a broad, generic construct (MHL) and PoDLi is perhaps too specific for a relationship between the two instruments. Future studies may assess the correlation between the C-PoDLiS and other psychometrically sound instruments having similar constructs, such as the 22-item Depression Literacy scale (Fonseca et al., 2017), to further verify the concurrent validity of C-PoDLiS. Moreover, the testing of predictive validity based on correlations between the scores of the C-PoDLiS and ATSPPH-SF revealed moderately positive significant correlations, indicating that perinatal women with a greater level of PoDLi will have a more favorable attitude toward professional psychological help-seeking. The findings further verified C-PoDLiS’s good validity and were consistent with previous studies in urban adults (Ho et al., 2018) and adolescent males (Clark et al., 2020). Nevertheless, this correlation study was not able to establish a causal relationship between the C-PoDLiS and the ATSPPH-SF. It will be worthwhile for future studies to address this unknown by investigating whether PoDLi promotion strategies can also improve attitudes toward PPD and its treatment.

Some important limitations should be acknowledged. First, although we tried to control the biases of our results by expanding the sample size and distributing questionnaires by well-trained researchers, the generalizability of the results of this study might still be threatened, as the participants were recruited from the Han ethnic group at a single tertiary hospital in the southeast coastal area of China, and more than half of the participants had a monthly household income above the national average and were well educated with a specialty/bachelor’s degree or above. Future research should include more diverse samples from various regions of China covering perinatal women of different ethnics, incomes, and educational levels to guarantee adequate representation and generalizability of this instrument in the entire country. Second, this validation study was performed on the basis of classical test theory, so the conclusion on the quality of the instrument’s measurement properties were population-dependent. It will be beneficial to conduct rasch analysis based on the non-parametric item response theory in future research, which allows conclusion to be drawn independently from the tested population (Nguyen et al., 2022), to provide additional important information on C-PoDLiS psychometric properties. Third, it is difficult for us to adequately compare our findings with the other relevant studies (Mirsalimi et al., 2020; Hairol et al., 2021; Poreddi et al., 2021) for the reason that, except for the study of developing the original version of PoDLiS (Mirsalimi et al., 2020), none of the others (Hairol et al., 2021; Poreddi et al., 2021) had performed formal evaluation of the psychometric properties of PoDLiS with Cronbach’s α reported only. As such, future studies need to evaluate and report the psychometric properties of the PoDLiS formally, which can enable the comparison between different versions of the PoDLiS and promote the popularization of the scale internationally. Finally, the cross-sectional study design precluded the explanation of causal relationships among variables as well as the assessment of the responsiveness of the C-PoDLiS, so longitudinal research will be needed for further validation.

This study has several potential implications for academic and clinical practice. First, on the individual level, the assessment of PoDLi from a self-evaluated perspective may raise perinatal women’s awareness of their mental health status, which can in turn promote them to utilize professional help-seeking when necessary. Moreover, at the academic level, C-PoDLiS paves the way for healthcare professionals to further conduct studies with regard to PoDLi and its influencing factors. Their findings will be helpful in better understanding specific elements of PoDLi that are lacking among Chinese perinatal women as well as identifying individuals with poor PoDLi levels, which lays the foundation for developing more effective and targeted interventions to improve the PoDLi level. At the same time, medical staff can use C-PoDLiS to evaluate the effectiveness of intervention programs, thereby adjusting the intervention strategies timely according to the evaluated results to maximize the intervention effects. Furthermore, on the national level, C-PoDLiS can inform the decision-making process for public health officials and can serve as a reference tool for the government and stakeholders to establish affordable mental health policies, as it is highly understandable and easily administered and can penetrate all layers of the community. More importantly, on the global level, this study provided the world with the data of PoDLi in China, enriching the evidence in the relevant field. However, additional studies are still required to verify the validity of the PoDLiS in other countries and different regions with different cultures, because a universal instrument can help with data aggregation and outcome comparisons across different studies and populations, as well as facilitate cross-border discussion of the PoDLi.

## Conclusion

Collectively, the 27-item, 6-dimension C-PoDLiS has satisfactory reliability and validity. It is an effective and concise instrument for evaluating the PoDLi level among perinatal women in China and consequently helping healthcare professionals to develop and implement effective and targeted interventions. Nevertheless, its application warrants further investigation in larger samples of Chinese perinatal women in different settings and areas.

## Data availability statement

The raw data supporting the conclusions of this article will be made available by the authors, without undue reservation.

## Ethics statement

The studies involving human participants were reviewed and approved by Ethics Committee of the Women’s Hospital, School of Medicine, Zhejiang University. The patients/participants provided their written informed consent to participate in this study.

## Author contributions

All authors made substantial contributions to conception and design, or acquisition of data, or analysis and interpretation of data, involved in drafting the manuscript or revised it critically for important intellectual content, gave final approval of the revision to be published, participated sufficiently in the work to take public responsibility for appropriate portions of the content, and agreed to be accountable for all aspects of the work in ensuring that questions related to the accuracy or integrity of any part of the work were appropriately investigated and resolved.

## Abbreviations

ATSPPH-SF: attitudes toward seeking professional psychological help short form scale
AVE: average variance extracted
CFA: confirmatory factor analysis
CFI: comparative fit index
COSMIN: Consensus-based Standards for the Selection of Health Status Measurement Instruments
CR: critical ratio
C-PoDLiS: Chinese version of postpartum depression literacy scale
EFA: exploratory factor analysis
GFI: goodness of fit
ICC: intraclass correlation coefficient
I-CVI: content validity index of items
IFI: incremental fit index
KMO: Kaiser–Meyer–Olkin
MHL: mental health literacy
MHLS: mental health literacy scale
PPD: postpartum depression
PoDLi: postpartum depression literacy
PoDLiS: postpartum depression literacy scale
RMSEA: root mean square error of approximation
S-CVI: scale-level content validity index
TLI: Tucker–Lewis index.
