# Supplementary material for: Cross-cultural adaptation and psychometric properties of the Chinese version of the postpartum depression literacy scale
Source: Front Psychol. 2022 Aug 9;13:966770. doi: 10.3389/fpsyg.2022.966770 (PMC9397490; doi:10.3389/fpsyg.2022.966770)
Supplement: Supplementary file 1 [file Data_Sheet_1.docx]

Supplementary Material

# Supplementary Tables

**Table S1.** The Chinese version of the postpartum depression literacy scale (C-PoDLiS).

| **Dimension** | **Item** | **Note** | **Whether the item is retained in the final version of C-PoDLiS** | **Response scale (5-point Likert scale)** |
| --- | --- | --- | --- | --- |
| Dimension 1：产后抑郁识别能力(Recognition abilities of postpartum depression) | Item 1：莫名的过度悲伤和流泪可能属于产后抑郁的症状(Inexplicable sadness and tears may be symptoms of postpartum depression) |  | Retained | 'strongly disagree' to 'strongly agree' |
|  | Item 2：睡眠过多或失眠可能属于产后抑郁的症状(Sleeping too much or insomnia may be a symptom of postpartum depression) |  | Retained | 'strongly disagree' to 'strongly agree' |
|  | Item 3：食欲不振或吃太多可能属于产后抑郁的症状(Loss of appetite or eating too much may be a symptom of postpartum depression) |  | Retained | 'strongly disagree' to 'strongly agree' |
|  | Item 4：对大多数事情失去兴趣或乐趣可能属于产后抑郁的症状(Lack of interest or pleasure in most things may be a postpartum symptom) |  | Retained | 'strongly disagree' to 'strongly agree' |
|  | Item 5：产后抑郁会对记忆力和注意力造成影响(Postpartum depression can affect memory and concentration) |  | Retained | 'strongly disagree' to 'strongly agree' |
|  | Item 6：产后抑郁的症状和体征至少会持续2周(The symptoms and signs of postpartum depression will last for at least 2 weeks) |  | Retained | 'strongly disagree' to 'strongly agree' |
| Dimension 2：产后抑郁相关信息的查询和判断能力(Ability to seek and judge information related to postpartum depression) | Item 28：我知道如何通过各种渠道（例如电脑、手机、看医生）获得产后抑郁相关信息(I know how to obtain information about postpartum depression through various channels (such computer, cellphone, or seeing a doctor)) | After being merged | Retained | 'strongly disagree' to 'strongly agree' |
|  | Item 29：我能判断广播和电视上产后抑郁相关信息的准确性(I can judge the accuracy of information about postpartum depression on radio and television) |  | Retained | 'strongly disagree' to 'strongly agree' |
|  | Item 30：我能判断互联网上产后抑郁相关信息的准确性(I can judge the accuracy of information related to postpartum depression on the Internet) |  | Retained | 'strongly disagree' to 'strongly agree' |
|  | Item 31：我能判断朋友和家人提供的产后抑郁相关建议的准确性(I can judge the accuracy of advices provided by friends and family related to postpartum depression) |  | Retained | 'strongly disagree' to 'strongly agree' |
| Dimension 3：对于促进产后抑郁认知或适当求助行为的态度(Attitudes towards promoting recognition of postpartum depression or appropriate help-seeking behaviors) | Item 22†：虽然有产后抑郁专科门诊，但我对它不太信任(Although there are specialist clinics for postpartum depression, I have little faith in it) |  | Retained | 'strongly disagree' to 'strongly agree' |
|  | Item 23†：我宁愿忍受产后抑郁，也不愿经受精神科治疗的痛苦(I would rather endure postpartum depression than go through painful psychiatric treatment) |  | Retained | 'strongly disagree' to 'strongly agree' |
|  | Item 24†：患有产后抑郁的女性大多有暴力倾向(Most women with postpartum depression are violent) |  | Retained | 'strongly disagree' to 'strongly agree' |
|  | Item 25†：最好避开产后抑郁患者，这样你就不会得产后抑郁(It's best to avoid patients with postpartum depression so that you won't have postpartum depression) |  | Retained | 'strongly disagree' to 'strongly agree' |
|  | Item 26†：如果我得了产后抑郁，我不会告诉任何人(If I have postpartum depression, I won't tell anyone) |  | Retained | 'strongly disagree' to 'strongly agree' |
|  | Item 27†：我担心家人或朋友对我去看心理医生或精神科门诊有看法(I'm worried about what my family and friends think of me for visiting to a psychologist or psychiatric clinic) |  | Retained | 'strongly disagree' to 'strongly agree' |
| Dimension 4：自我照护活动知识和信念(Knowledge and belief in self-care activities ) | Item 12：体育锻炼能有效预防或治疗产后抑郁(Physical exercise can effectively prevent or treat postpartum depression) |  | Retained | 'strongly disagree' to 'strongly agree' |
|  | Item 13：请伴侣和家庭成员帮忙照顾宝宝和分担家务等有助于预防或治疗产后抑郁(Asking partners and family members to take care of the baby and share housework will contribute to prevent or treat postpartum depression) |  | Retained | 'strongly disagree' to 'strongly agree' |
|  | Item 14：宗教活动、祷告和朝圣有助于预防或治疗产后抑郁(Religious activities, prayers and holy shrine visits are helpful for the prevention or treatment of postpartum depression) |  | Deleted | 'strongly disagree' to 'strongly agree' |
|  | Item 15：休闲娱乐活动（如听音乐、看电影、亲友聚会等）有助于预防或治疗产后抑郁(Leisure and entertainment activities (such as listening to music, going to movies, gathering with friends and families, etc.) are helpful in preventing or treating postpartum depression) | Newly added | Retained | 'strongly disagree' to 'strongly agree' |
|  | Item 16：均衡饮食有助于预防或治疗产后抑郁(Having a balanced diet is helpful in preventing or treating postpartum depression) |  | Retained | 'strongly disagree' to 'strongly agree' |
|  | Item 17：良好的睡眠有助于预防或治疗产后抑郁(Good sleep is helpful in preventing or treating postpartum depression ) |  | Retained | 'strongly disagree' to 'strongly agree' |
| Dimension 5：风险因素和致病原因(Knowledge of risk factors and causes) | Item 7：您认为产后抑郁由基因或遗传因素引起的可能性有多大？(How likely is it that postpartum depression may be caused by genetic or hereditary problems?) |  | Deleted | 'not likely at all' to 'very likely' |
|  | Item 8：您认为产后抑郁由压力性生活事件（如亲人去世或离婚）引起的可能性有多大？(How likely is it that postpartum depression may be caused by stressful life events (such as the death of a loved one or divorce)?) |  | Retained | 'not likely at all' to 'very likely' |
|  | Item 9：您认为产后抑郁由社会支持缺乏（如缺乏亲密伴侣支持）引起的可能性有多大？(How likely is it that postpartum depression may be caused by the lack of social support (such as the lack of intimate partner support)?) |  | Retained | 'not likely at all' to 'very likely' |
|  | Item 10：您认为产后抑郁由既往抑郁病史引起的可能性有多大？(How likely is it that postpartum depression may be caused by a previous history of depression?) |  | Retained | 'not likely at all' to 'very likely' |
|  | Item 11：您认为产后抑郁由激素失衡引起的可能性有多大？(How likely is it that postpartum depression may be caused by hormone imbalance?) |  | Retained | 'not likely at all' to 'very likely' |
| Dimension 6：有关可获得的专业帮助知识和信念(Knowledge and beliefs about professional help available) | Item 18：由专业人员（心理咨询师和精神科医生等）提供的产后抑郁治疗(如心理咨询)是有效的(Treatment of postpartum depression (such as psychological counseling) provided by professionals (psychological counselors and psychiatrists, etc.) can be effective) | After being merged | Retained | 'strongly disagree' to 'strongly agree' |
|  | Item 19†：抗抑郁药具有成瘾性(Antidepressants are addictive) |  | Deleted | 'strongly disagree' to 'strongly agree' |
|  | Item 20†：抗抑郁药会导致大脑损伤(Antidepressants can cause brain damage) |  | Deleted | 'strongly disagree' to 'strongly agree' |
|  | Item 21：抗抑郁药可有效治疗产后抑郁(Antidepressants are effective in treating postpartum depression) | Newly added | Retained | 'strongly disagree' to 'strongly agree' |
| C-PoDLiS, Chinese version of the postpartum depression literacy scale; †: means reverse-scored items; The total score for PoDLiS as well as scores for each dimension are calculated by adding up the coded values and then dividing them into the number of items, ranging from 1 to 5. | | | | |

**Table S2.** The exploratory factor analysis results of the C-PoDLiS.

| **Dimension/Item number** | Pattern Matrix | | | | | |  | Structure Matrix | | | | | |
| --- | --- | --- | --- | --- | --- | --- | --- | --- | --- | --- | --- | --- | --- |
|  | Factor 1 | Factor 2 | Factor 3 | Factor 4 | Factor 5 | Factor 6 |  | Factor 1 | Factor 2 | Factor 3 | Factor 4 | Factor 5 | Factor 6 |
| **Dimension 1** |  |  |  |  |  |  |  |  |  |  |  |  |  |
| Item 1 | **0.632** | -0.113 | 0.018 | 0.111 | 0.107 | 0.044 |  | **0.717** | 0.014 | 0.141 | 0.392 | 0.38 | 0.223 |
| Item 2 | **0.807** | 0.062 | -0.079 | -0.011 | -0.008 | -0.105 |  | **0.775** | 0.126 | -0.016 | 0.288 | 0.253 | 0.062 |
| Item 3 | **0.828** | 0.029 | -0.016 | -0.034 | -0.089 | -0.035 |  | **0.775** | 0.088 | 0.029 | 0.27 | 0.203 | 0.116 |
| Item 4 | **0.718** | -0.084 | 0.01 | -0.041 | 0.134 | 0.1 |  | **0.765** | 0.023 | 0.126 | 0.305 | 0.403 | 0.267 |
| Item 5 | **0.603** | -0.038 | 0.07 | -0.005 | 0.074 | 0.146 |  | **0.663** | 0.065 | 0.174 | 0.312 | 0.35 | 0.3 |
| Item 6 | **0.594** | 0.029 | 0.097 | 0.081 | -0.122 | 0.093 |  | **0.614** | 0.113 | 0.156 | 0.337 | 0.187 | 0.233 |
| **Dimension 2** |  |  |  |  |  |  |  |  |  |  |  |  |  |
| Item 28 | -0.019 | **0.705** | 0.011 | 0.002 | 0.088 | 0.058 |  | 0.106 | **0.728** | 0.083 | 0.239 | 0.225 | 0.183 |
| Item 29 | -0.049 | **0.891** | -0.006 | 0.047 | -0.001 | 0.032 |  | 0.074 | **0.903** | 0.053 | 0.282 | 0.163 | 0.164 |
| Item 30 | -0.022 | **0.926** | -0.017 | -0.008 | -0.028 | 0.041 |  | 0.072 | **0.922** | 0.027 | 0.238 | 0.132 | 0.162 |
| Item 31 | 0.033 | **0.861** | 0.041 | -0.023 | 0.003 | -0.01 |  | 0.12 | **0.859** | 0.084 | 0.238 | 0.171 | 0.126 |
| **Dimension 3** |  |  |  |  |  |  |  |  |  |  |  |  |  |
| Item 22 | 0.013 | 0.047 | **0.656** | -0.043 | 0.097 | 0.189 |  | 0.145 | 0.117 | **0.71** | 0.213 | 0.34 | 0.318 |
| Item 23 | -0.213 | -0.039 | **0.613** | 0.09 | 0.115 | 0.209 |  | -0.03 | 0.047 | **0.679** | 0.23 | 0.296 | 0.308 |
| Item 24 | 0.056 | 0.052 | **0.646** | -0.178 | -0.145 | 0.03 |  | 0.006 | 0.022 | **0.574** | -0.032 | 0.026 | 0.072 |
| Item 25 | 0.158 | -0.074 | **0.73** | -0.083 | -0.028 | -0.033 |  | 0.172 | -0.05 | **0.709** | 0.115 | 0.202 | 0.081 |
| Item 26 | 0.017 | -0.074 | **0.781** | 0.142 | -0.051 | -0.163 |  | 0.092 | -0.024 | **0.77** | 0.251 | 0.186 | -0.019 |
| Item 27 | -0.043 | 0.123 | **0.741** | 0.033 | 0.022 | -0.207 |  | 0.023 | 0.14 | **0.724** | 0.176 | 0.212 | -0.064 |
| **Dimension 4** |  |  |  |  |  |  |  |  |  |  |  |  |  |
| Item 12 | 0.071 | 0.104 | -0.063 | **0.756** | -0.057 | -0.104 |  | 0.342 | 0.293 | 0.093 | **0.752** | 0.205 | 0.1 |
| Item 13 | 0.091 | 0.039 | -0.056 | **0.83** | -0.015 | -0.012 |  | 0.422 | 0.272 | 0.144 | **0.857** | 0.294 | 0.219 |
| Item 15 | -0.062 | -0.071 | 0.058 | **0.834** | -0.016 | 0.121 |  | 0.299 | 0.172 | 0.259 | **0.829** | 0.284 | 0.321 |
| Item 16 | 0.16 | 0.166 | 0.017 | **0.533** | 0.044 | -0.104 |  | 0.392 | 0.325 | 0.164 | **0.637** | 0.298 | 0.109 |
| Item 17 | -0.111 | -0.128 | -0.026 | **0.739** | 0.056 | 0.152 |  | 0.23 | 0.097 | 0.171 | **0.711** | 0.278 | 0.313 |
| **Dimension 5** |  |  |  |  |  |  |  |  |  |  |  |  |  |
| Item 8 | 0.015 | -0.004 | -0.087 | 0.03 | **0.844** | -0.113 |  | 0.311 | 0.136 | 0.158 | 0.277 | **0.805** | 0.092 |
| Item 9 | -0.091 | -0.032 | -0.004 | 0.091 | **0.835** | -0.052 |  | 0.245 | 0.125 | 0.25 | 0.319 | **0.812** | 0.154 |
| Item 10 | 0.02 | 0.075 | -0.024 | -0.156 | **0.809** | 0.161 |  | 0.301 | 0.202 | 0.215 | 0.19 | **0.809** | 0.332 |
| Item 11 | 0.126 | 0.037 | 0.082 | 0.014 | **0.644** | -0.125 |  | 0.36 | 0.156 | 0.274 | 0.285 | **0.697** | 0.084 |
| **Dimension 6** |  |  |  |  |  |  |  |  |  |  |  |  |  |
| Item 18 | 0.122 | 0.051 | -0.064 | -0.007 | -0.02 | **0.791** |  | 0.282 | 0.173 | 0.072 | 0.243 | 0.209 | **0.808** |
| Item 21 | 0.007 | 0.053 | -0.001 | 0.086 | -0.089 | **0.838** |  | 0.196 | 0.186 | 0.132 | 0.293 | 0.161 | **0.848** |
| % of the variance | **-** | **-** | **-** | **-** | **-** | **-** |  | 6.316 | 2.875 | 2.707 | 1.758 | 1.454 | 1.361 |
| Cumulative variance | **-** | **-** | **-** | **-** | **-** | **-** |  | 23.393 | 34.039 | 44.065 | 50.575 | 55.961 | 61.002 |
| C-PoDLiS, Chinese version of the postpartum depression literacy scale; Factor loadings with an absolute value > 0.450 are displayed in bold. | | | | | | | | | | | | | |
